# Learning stochastic process-based models of dynamical systems from knowledge and data

**DOI:** 10.1186/s12918-016-0273-4

**Published:** 2016-03-22

**Authors:** Jovan Tanevski, Ljupčo Todorovski, Sašo Džeroski

**Affiliations:** Jožef Stefan Institute, Jamova cesta 39, Ljubljana, 1000 Slovenia; Jožef Stefan International Postgraduate School, Jamova cesta 39, Ljubljana, 1000 Slovenia; University of Ljubljana, Gosarjeva ulica 5, Ljubljana, 1000 Slovenia

**Keywords:** Process-based modeling, Structural uncertainty, Dynamical systems, Stochastic models, Genetic regulatory networks, Compartmental epidemiological models

## Abstract

**Background:**

Identifying a proper model structure, using methods that address both structural and parameter uncertainty, is a crucial problem within the systems approach to biology. And yet, it has a marginal presence in the recent literature. While many existing approaches integrate methods for simulation and parameter estimation of a single model to address parameter uncertainty, only few of them address structural uncertainty at the same time. The methods for handling structure uncertainty often oversimplify the problem by allowing the human modeler to explicitly enumerate a relatively small number of alternative model structures. On the other hand, process-based modeling methods provide flexible modular formalisms for specifying large classes of plausible model structures, but their scope is limited to deterministic models. Here, we aim at extending the scope of process-based modeling methods to inductively learn stochastic models from knowledge and data.

**Results:**

We combine the flexibility of process-based modeling in terms of addressing structural uncertainty with the benefits of stochastic modeling. The proposed method combines search trough the space of plausible model structures, the parsimony principle and parameter estimation to identify a model with optimal structure and parameters. We illustrate the utility of the proposed method on four stochastic modeling tasks in two domains: gene regulatory networks and epidemiology. Within the first domain, using synthetically generated data, the method successfully recovers the structure and parameters of known regulatory networks from simulations. In the epidemiology domain, the method successfully reconstructs previously established models of epidemic outbreaks from real, sparse and noisy measurement data.

**Conclusions:**

The method represents a unified approach to modeling dynamical systems that allows for flexible formalization of the space of candidate model structures, deterministic and stochastic interpretation of model dynamics, and automated induction of model structure and parameters from data. The method is able to reconstruct models of dynamical systems from synthetic and real data.

## Background

Most systems in biology exhibit dynamical behavior. Their properties change as a function of time and space in a complex manner. Considering a dynamical biological system to be a well-stirred mixture of its constituents, the most commonly used mathematical model of its dynamics takes the form of a system of coupled ordinary differential equations, treating the entity properties as continuous and assuming they evolve deterministically through time. However, the deterministic nature of ordinary differential equations renders them inadequate for systems with a small number of copies (only few orders of magnitude above one) of its constituents. Furthermore, ordinary differential equations fail to account for the underlying stochasticity of natural systems [1, 2]. In molecular systems, stochastic fluctuations are responsible for the divergence in phenotype and genetic activities [3–5]. In such cases, models based on stochastic kinetics are more suitable, as they allow for treating of the modeled systems as either discrete or continuous in terms of the properties of the observed entities and stochastic in terms of the reactions between them.

Establishing a deterministic or a stochastic model of an observed biological system is an omnipresent and often complex, tedious task. This task comprises the two subtasks of structure identification, i.e., selecting an appropriate model structure, and parameter estimation, i.e., determining values of the model parameters that, together with the selected structure, lead to accurate reconstruction of the observed system behavior. While many existing approaches integrate methods for simulation and parameter estimation of a single model, only few of them provide support for the task of structure identification [6, 7]. In this paper, we design and implement a computational tool that can deal with uncertainty in both model structure and the values of model parameters for both deterministic and stochastic models. The central component of our tool is the process-based modeling formalism that allows for modular, compositional specification of the space of candidate model structures.

**Fig. 1:**
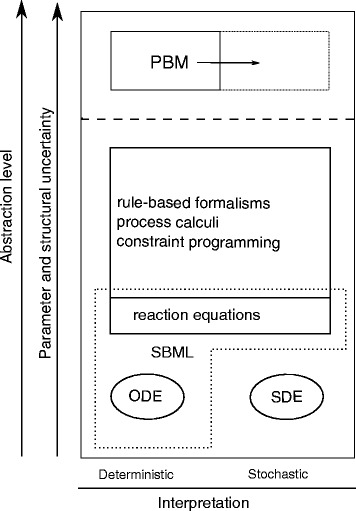
The relation of the process-based modeling formalism to other formalisms used for modeling dynamical systems in biology

Figure 1 puts the process-based modeling formalism in the context of existing formalisms used for modeling biological systems. The figure sorts (along the vertical axis) different formalisms according to their abilities to specify uncertainty with regard to the model parameter values and uncertainty with regard to the model structure. The vertical axis also refers to model specifications at different abstraction levels, from low-level model implementation to high-level model specification [8]. The horizontal axis refers to the possibilities of model interpretation: some of the formalisms are focused on deterministic, some on stochastic, while the third group of formalisms allows for both deterministic and stochastic model interpretation.

The formalisms of differential equations allow for encoding models with all the details needed for their execution, i.e., simulation of the behavior of the corresponding dynamical systems. Ordinary differential equations are limited to deterministic model interpretation, while stochastic differential equations are used for stochastic modeling. Differential equations are models at the lowest abstraction level, where every detail has to be fully specified and are used to encode a single model; on their own, they allow neither for parameter nor structural uncertainty.

At a higher abstraction level, the models in the domain of biology are often casted in the formalism of reaction equations. Following this formalism, the biological system is described as a reaction network. When coupled with appropriate kinetic rates, the model defines a network of possible transitions between system states. Reaction equations allow for both deterministic and probabilistic interpretation stemming from the propensity of each reaction [9].

The systems biology markup language, SBML [10], is a standard modeling formalism in system biology. It allows for encoding and exchange of individual models based on ordinary differential equations or reaction equations. Like equation-based formalisms, SBML focuses on encoding a single model structure with parameter uncertainties and does not support the specification of structural uncertainties.

Furthermore, a number of formalisms have emerged that deal with the issue of combinatorial complexity, i.e., the exponential complexity of the space of the combinations of elementary interactions between the entities observed in a given biological system. These formalisms allow for specifying rules (constraints) that limit the space of potential interactions between entities based on their properties. Note that the encoded constraints do not address the issue of structural uncertainty: Their application in the context of a given observed system leads to a single model structure. There are several classes of such formalisms.

The first group of rule-based (also referred to as interaction-based) languages, most notably BioNetGen [11] and kappa [12], define the constituent entities of a system at the level of objects with different properties. The network of interactions between the system entities is implicitly described by a set of rules that transform properties or create new entities (by forming complexes of existing entities). By defining the rules directly on the properties, the rule-based modeling approach efficiently deals with the problem of combinatorial complexity, which may arise when modeling protein-protein networks within complex signalization pathways. The rules are encoded using formalisms based on reaction equations.

The second group of agent-based formalisms, which includes process algebras [13, 14], model individual entities as agents in a complex system that act according to a set of predefined rules for communication with other agents. The process algebras describe the behavior of each agent through processes describing the inter-agent communications via different channels. A biological system described using process calculi is treated as a constrained distributed system of communication. This formal description allows for more detailed representation of the basic principles of interaction. Examples of process algebra extensions that have been adapted to and are being used in the domain of biology are the stochastic pi-calculus [15], Bio-PEPA [16] and beta binders [17].

Related to the process algebras group, the formalisms in the third group are based on constraint programming [18]. In contrast to the process calculi, the constraint programming approaches allow for defining interactions not only through specific communication channels, but by concurrently posting global constraints on the properties of the agent entities.

The limitation that is common to all aforementioned formalisms is that they can not properly represent the structural uncertainty. Uncertainty in parameter values is typically addressed by various formalism extensions that are complementary to the computational tools that offer support for them. COPASI [19] is an example of a such a tool that allows for introducing uncertainties in model parameter values and performing parameter estimation for models based on equations. The MathWorks SimBiology toolbox [20] is a proprietary software for modeling and analysis of dynamical systems in biology providing features similar to the ones of COPASI. Both tools provide a range of methods for the analysis of models (e.g., sensitivity and identifiability of model parameters), but do not provide computational methods for addressing structural uncertainty; users can only perform manual comparative analysis of different model structures.

Network inference methods [21] explicitly address structural uncertainty: most often, given gene expression data, the methods seek for a network of interactions between the observed genes. Since these methods focus on the structure of the observed network of interactions, they seldom deal with the reconstruction of the dynamical behavior of the observed system. Several methods are exception to this general rule and cast the reconstructed networks into the formalism of ordinary differential equations [21–24]. In contrast to the process-based modeling approach presented in this paper, these methods are limited to deterministic models. Furthermore, these methods follow the assumption that the same interaction dynamics applies to all of the network interactions: the process-based modeling formalism can encode different classes of model structures (interactions/processes) with different assumptions about the interaction dynamics. Finally, when it comes to constraining the space of possible model structures, some methods employ data-driven heuristics [22, 25], while some of them additionally limit the search for plausible structures based on the interactions already documented in the literature [22, 24]. The method by Wahl et al. [22] also allows for user-defined Boolean constraints specifying implausible network interactions.

Finally, ABC-SysBio [6] is most closely related to the process-based modeling approach presented here. It builds on SBML and addresses structural uncertainty by allowing the user to explicitly enumerate the alternative model structures. The process-based modeling formalism the we propose addresses exactly this limitation of the existing formalisms, i.e., the ability to properly address structural uncertainty. It allows for modular and flexible specification of the space of candidate model structures to be considered in the modeling process. Instead of specifying a fixed list of candidates, like ABC-SysBio [6], our formalism allows users to specify model components, which are then used in a compositional modeling setting, where combinations of components correspond to candidate model structures. Thus, we approach the structure identification task as a search problem [26], where we search for the most appropriate combination of model components.

This paper builds upon our previous work on inductive process-based modeling that combines knowledge and data to automatically build explanatory models of dynamical systems [27–30]. While inductive process-based modeling has been successfully applied to modeling tasks in the domain of systems biology [7, 31, 32], its scope has been limited to building deterministic models of dynamical systems cast as ordinary differential equations.

Here, we extend the scope of the process-based modeling formalism to models cast as reaction equations, hence the arrow in the top-right corner of Fig. 1. In this way, we combine the benefits of process-based modeling (in terms of addressing structural uncertainty) with the benefits of different model interpretations (including the stochastic interpretation). Finally, the formalism is implemented within a computational tool ProBMoTs for automated induction of models that combines domain knowledge represented in our formalism with measurements of the observed system behavior.

In the remainder of the paper, we first introduce the process-based modeling formalism, its extensions towards handling stochastic models of biochemical systems and the computational tool for process-based modeling, ProBMoTs. We present then two examples of use of the proposed computational tool, i.e. modeling gene regulatory networks and modeling the spread of pathogens, illustrating the use of the proposed tool and evaluating its utility. Finally, we discuss the results of the evaluation, put them in the context of existing work and outline directions for further research.

## Methods

In this section, we introduce the notion of process-based models and a formalism for their representation. We illustrate the formalism use on an example of encoding knowledge for modeling gene regulatory networks and a process-based model of a specific network, the repressilator [33]. We then introduce methods for inducing process-based models from knowledge and data by selecting appropriate model structures and parameters.

### Process-based modeling

Scientists often describe dynamical systems in terms of processes that govern the system dynamics and the entities involved in the processes^1^. Following this high-level model description, modelers assign lower-level detailed equation-based specifications of the dynamics to individual processes and combine them into a system of coupled differential equations. The differential equations can be in turn used to simulate the behavior of the observed system or to extrapolate the simulation and predict future system behavior. However, by transforming the high-level model description into equations, its explanatory power is lost, since the equations fail to reveal (in an accessible manner) the structure of the observed system in terms of the interacting entities and processes.

Process-based modeling (PBM) clearly relates a high-level model description (entities and processes), that carries significant explanatory power, and a lower-level mathematical model (equations), that allows for simulation and prediction. To build process-based models, we first formalize the modeling knowledge by establishing templates of generic (template) entities that appear in the generic (template) processes that govern the dynamics of systems in the particular domain. Each process-based model then refers to these template components and instantiates them into specific components of the studied system.

Existing process-based formalisms rely on a coarse description of dynamics, based on fragments of differential equations. The formalism introduced in this paper relies on reaction equations, which are closer to the basic principles of system biology and are more comprehensible to biologists. A reaction equation *R*_*s*_→*P*_*s*_ [*rate*] specifies a set of reactants *R*_*s*_ and a set of products *P*_*s*_, as well as the reaction *rate*. Reaction equations are a powerful and flexible formalism for modeling the temporal evolution of dynamical systems.

#### Representation of modeling knowledge

Table 1 provides an example library of template components for modeling gene regulatory networks. It includes a template entity *gene*, whose instances represent nodes in gene regulatory networks. We assume gene entities to represent protein-coding genes and describe them using five numerical properties. The variable properties (*vars* section of the entity specification) denote two gene properties that change through time: *Pmol* is the number of encoded protein molecules and *mRNAmol* is the number of mRNA transcripts. The other three properties do not change over time; they denote the constant kinetic rates of the uncontrolled gene expression *alpha0*, the translation of mRNA into proteins and their degradation *beta*, as well as the mRNA molecules degradation *delta*.

**Table 1:** Templates of entities and processes for modeling gene regulatory networks. The template entity *gene* typifies network nodes, while the process templates represent gene regulation, as well as translation and protein degradation processes. The empty set symbol *∅* denotes the absence of reactants or products

	

Furthermore, the library specifies templates for modeling the processes of gene interaction, gene translation into proteins, and protein degradation. The *degradation* template specifies two reaction equations that correspond to the degradation of the encoded protein molecules with the kinetic rate of *g.beta* (i.e., the degradation kinetic rate for the particular gene *g*) and the degradation of the mRNA molecules with the rate of *g.delta*. Similarly, the *translation* process integrates the reaction equations of the gene transcription to mRNA and the mRNA translation to protein molecules.

Finally, the *regulation* process template represents gene interactions via their protein products. It has two mutually exclusive alternatives of *activation* and *inhibition*. The first corresponds to the case where one gene increases the transcription rate of the other, while the second alternative models repression, where one gene decreases the transcription rate of the other by binding the source gene protein to the promoter region of the repressed gene. In both cases, the reaction rate (specified between the brackets) is modeled using a Hill function, derived as a steady-state approximation of the biochemical kinetics [34].

The templates from Table 1 represent generic knowledge on modeling gene regulatory networks. They can be instantiated to entities and processes of an arbitrary network model. Note the hierarchical structure of the *regulation* template process: it constrains the space of instantiations by rendering the two subordinate templates of *activation* and *inhibition* mutually exclusive. This reflects the simple fact that only one regulation type applies to a given pair of genes. In the following, we will illustrate the use of this knowledge for modeling a simple regulatory network.

#### Process-based models

**Fig. 2:**
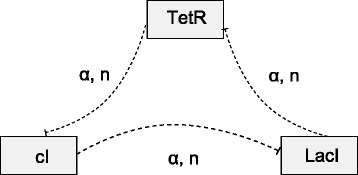
Graphical representation of the repressilator gene regulatory network

The repressilator [33] is a regulatory network of three genes interacting in a single feedback loop of inhibitions as depicted in Fig. 2. The repressilator is a synthetic network designed to exhibit a stable oscillatory behavior. Its in-vivo implementation in *E. coli* has been proven to exhibit the desired behavior. The three genes involved are TetR, often used for fine regulation in synthetic gene networks, and two repressor genes, cI and LacI.

Using the domain knowledge for modeling gene regulatory networks from Table 1, we can establish a process-based model of the repressilator, presented in Table 2. It provides a high-level representation akin to the graphical network layout depicted in Fig. 2, where entities correspond to network nodes, and processes are represented by arcs. The model does not give details about the particular modeling choices for degradation, translation and inhibition, since they are inherited from the corresponding process templates. Each entity specifies the boundary conditions for the variables (declarations of the *initial* value) and the parameter values, while each process specifies the involved entities and the parameter values. Note, for example, the value assignments for the parameters *alpha* and *n* in the inhibition processes.

**Table 2:** A process-based model of the repressilator built using the templates for modeling gene regulatory networks from Table 1

	

The process-based model retains the understandability of the graphical model representation and provides a clear, high-level insight into the structure of the studied system. At the same time, by using the detailed knowledge of the reaction equations encoded in the templates, we can automatically translate the high-level description into a mathematical model and use it for simulation and analysis. Consider the process *translation1* in Table 2: By combining it with the template *translation* from Table 1, we instantiate a set of two reaction equations modeling the uncontrolled transcription of *TetR* to mRNA (* ∅ -> TetR.mRNAmol* with a kinetic rate of *TetR.alpha0 = 0*) and the translation of the mRNA to the TetR protein molecules (*TetR.mRNAmol* -> *TetR.Pmol + TetR.mRNAmol* with a kinetic rate of *TetR.beta = 9.75*).

Table 3 presents the mathematical model of the repressilator which includes the above two reaction equations, as well as all other reaction equations obtained by combining the processes in Table 2 with their corresponding templates from Table 1. The model is simulated by calculating the state of the system *x*(*t*), a vector of the number of molecules of each reactant at time *t*. The repressilator state includes six variables: *TetR.Pmol*, *TetR.mRNAmol*, *LacI.Pmol*, *LacI.mRNAmol*, *cI.Pmol* and *cI.mRNAmol*. In any given state *x*, we can calculate the propensity, i.e., the probability that the reaction *R*_*j*_ will be active in the infinitesimal time interval [*t, t*+*dt*), using the formula *a*_*j*_(*x*)=*c*_*j*_*h*_*j*_(*x*)*dt*, where *c*_*j*_ denotes the reaction rate and *h*_*j*_(*x*) denotes the number of distinct combinations of reactant molecules in state *x*.

**Table 3:** List of reaction equations stemming from the process-based model of the repressilator from Table 2

	

The evolution of the probability *P*(*x,t*|*x*_0_,*t*_0_) that the system is in a state *x* at a given time *t*, given the initial state *x*_0_ at time *t*_0_, can be then defined using the following ordinary differential equation (also known as the Master Equation) [35]:

(1) $${} \begin{aligned} \frac{\partial}{\partial t}P&(x,t|x_{0},t_{0})\\[-6pt] &\!= \sum_{j=1}^{M}[\!a_{j}(x - \nu_{j})P(x - \nu_{j},t|x_{0},t_{0}\!) - a_{j}(x)P(x,t|x_{0},t_{0})\!], \end{aligned}  $$

for *dt*→0, where *ν*_*j*_ is a vector specifying the changes of the number of reactant molecules after the reaction *R*_*j*_. We can then model the system dynamics using coupled differential equations, where each equation models the probability that the system state equals a unique combination of values of the state variables *x*.

For real biological systems, the Master Equation is too complex to be solved analytically or numerically. To this end, alternative approaches to estimating the exact or approximate probabilities have been developed. One of the most popular exact approaches is based on Monte Carlo sampling and is known as the Stochastic Simulation Algorithm (SSA) proposed by Gillespie, where others include the Gibson-Bruck method of next reaction and the class of *τ*-leaping methods [9].

If we assume that the propensity does not significantly change in infinitesimal time intervals and that the expected number of firings of each reaction is significantly large (i.e., the number of reactant molecules is large compared to the probability rate constant), we can derive the Langevin Equation. It represents a mathematical model of the reaction equations cast in terms of coupled Itō stochastic differential equations [36]. These stochastic differential equations can further be reduced to ordinary differential equations, under the assumption that we observe a negligible amount of noise in a system with a large number of reactants.

**Fig. 3:**
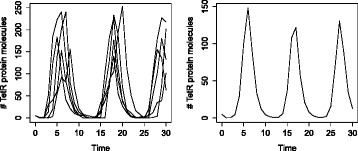
Stochastic and deterministic simulation of the number of TetR protein molecules using the process-based model of the repressilator

Thus, from a process-based model, we can automatically infer the reaction equations and then simulate them using the Gillespie algorithm or its improvements [9]. Alternatively, we can transform them to a system of ordinary differential equations. Figure 3 shows the simulated trajectories of the number of *TetR* molecules obtained by simulating the reaction equations (left-hand side) and the system of ordinary differential equations (right-hand side) inferred from the process-based model of the repressilator.

To summarize, process-based models have four important properties that make them particularly suitable for modeling dynamical systems. First, they retain the *understandability* and explanatory power of graphical model representations by providing clear insight into the structure of the observed system. At the same time, they inherit the *utility* of mathematical models for simulation and analysis of system behavior. Third, process-based models provide *general* model descriptions that support both stochastic and deterministic approaches to modeling, simulation and analysis. The fourth property is the *modularity* provided by the knowledge representation formalism: the templates can be instantiated into a number of model components. This last property is particularly relevant for the algorithms that induce process-based models from data.

#### Inducing process-based models

The formalized knowledge on modeling gene regulatory networks brings another benefit. It represents a source of constraints that limit the space of candidate model structures to be explored when modeling a particular gene regulatory network. Consider the repressilator model again and assume that we are only provided with information that it involves the three genes of *TetR*, *LacI* and *cI*. Now we can infer all the instances of the process templates from Table 1: the *degradation* process template that involves one gene, leads to three process instantiations, one for each gene. Similarly, the *translation* template leads to three processes. Finally, each pair of genes results in one instance of the activation and one instance of the *inhibition* template. Thus, for the three repressilator genes, we obtain six instances of the *activation* and six instances of the *inhibition* template. In sum, the three repressilator genes lead to 18 process instances.

Each of the process instances represents a valid model component. Following a naïve approach, one can consider any subset of components as a legitimate model structure, which yields 2^18^=262,144 candidates. However, these include many implausible models, e.g., ones that do not include gene translation for some of the genes. To avoid implausible models, the inductive process modeling approach relies on the use of constraints that limit the ways model components are combined. For example, a constraint ruling out models that do not include translation and degradation processes for all the genes, reduces the search space to 2^12^=4096 candidates. Furthermore, the constraint specifying the mutual exclusivity of the activation and inhibition processes for a given ordered pair of gene entities further reduces the number of candidates to 3^6^=729 (for each of the six possible pairs of repressilator genes, we consider three modeling alternatives: absence of regulatory influence; activation; and inhibition).

The constraints discussed above can be classified in two groups. First, the mutual exclusivity of the activation and inhibition processes is specified in the domain knowledge library shown in Table 1. Second, the constraint ruling out models that do not include translation and degradation of individual gene/protein are defined at the level of process instances. The constraints from the second group are specified in the incomplete model, which is one of the inputs to our software tool ProBMoTs. One such incomplete model is depicted graphically in Fig. 5 and shown in Table 4. The lower part of the table specifies that the model must include both a translation and a degradation process for each of the three genes/proteins (note the mandatory qualifier in the process specifications). Figure 5 (and the upper part of Table 4) specifies the three modeling alternatives for each of the six possible pairs of genes.

**Table 4:** An incomplete process-based model of a gene regulatory network specifying the model structures as depicted in Fig. 5

	

Finally, the inductive process-based modeling approach validates each candidate model structure by matching its simulation against the observed system behavior. In order to simulate the model (and assess its quality), we first have to determine the values of its constant parameters. To this end, we employ parameter estimation and find parameter values that lead to a model reproducing the observed behavior as closely as possible. We formulate the parameter estimation task as an optimization problem: We aim at minimizing an objective function that measures the goodness of fit of the model simulation to the observed behavior using the maximum-likelihood estimator [37].

The algorithm for inducing process-based models, presented in Table 5, puts together the components outlined above. Its input is a library of template entities and processes, such as the one presented in Table 1, the specific entity instances observed in the system at hand, a set of constraints that limit the way we combine components into models, and time-series data comprising measurements of the system variables/outputs of observed system. The algorithm first instantiates the templates from the library using the entities of the observed system into a set of model components. Then, taking into account the constraints, the algorithm enumerates the plausible combinations of components as candidate model structures. For each model structure, the algorithm performs parameter estimation that fits the model simulation against observed data. At output, the algorithm returns a list of models ranked with respect to their fit against the measured data.

**Table 5:** Top-level outline of the algorithm for inducing process-based models from knowledge and data

	

Different implementations of the induction algorithm make different design choices. In the following, we provide a brief overview of the different implementations: A detailed overview is given by Džeroski and Todorovski [7]. Lagramge 2.0 [38] transforms the library and constraints into a grammar that enumerates candidate model structures. IPM [28] takes a naïve approach and uses constraints on the number of components involved in the model to address combinatorial explosion. HIPM [29] encodes the constraints into a hierarchy of process templates and approaches enumeration as a combinatorial search problem. SCIPM [39] explicitly encodes the constraints and approaches the enumeration using constraint satisfaction methods. Finally, ProBMoT [40] extends HIPM with explicit constraints referring to the particular system at hand and meta-heuristic optimization methods for parameter estimation.

Note, however, that the above inductive process-based modeling approaches have limited their focus on inducing deterministic models cast as ordinary differential equations. ProBMoTs, our extension of ProBMoT presented in this paper that allows for inducing stochastic models of dynamical systems cast as reaction equations. The extension is based on the novel formalism for encoding a library of components that supports the specification of reaction equations as models of individual processes. ProBMoTs also integrates standard simulators for reaction equations [41].

Both ProBMoT and ProBMoTs are released as open-source software packages available for download at http://probmot.ijs.si^2^.

### Experimental setup and model selection

To evaluate the algorithm for inducing stochastic process-based models, we apply it to several problems of modeling dynamical behavior of biological systems at different scales. We consider two synthetic modeling problems from the domain of gene regulatory networks and two real modeling problems from the domain of epidemiology. In each domain, we first encode process-based knowledge for modeling dynamical systems. In this paper, our focus is limited to encoding domain knowledge in two domains, covering models on fundamentally different scales. Note, however, that the process-based modeling approach can be applied to other domains as well, given that modeling knowledge about the domain of interest is encoded as a library of entity and process templates. For example, when modeling metabolic networks, the central entity templates will represent enzymes and metabolites, while process templates would represent different metabolic reactions (with different kinetics), formulating different models of the dynamical interactions among them. For further examples of domain knowledge libraries for process-based modeling, we refer the reader to the ProBMoT web site. Second, for the synthetic modeling problems, we select a target model and simulate it to obtain a data set for inducing models. On the other hand, the real modeling problems come with data sets of measured system behavior. Third, for each modeling problem, we define an ordered list of plausible model structures *P*. For the synthetic problems, this list includes the target model only, while for the real modeling problems, it includes all the structures of the models that have been reported in the literature as plausible explanations of the measurements. Note that for all problems, the list of candidate models considered by the induction algorithm includes all the model structures from the list *P*.

To perform induction, for each modeling problem we run ProBMoTs using the corresponding modeling knowledge (including the constraints) and the data set as inputs. Recall that the modeling knowledge defines the space of candidate model structures. The values of the model parameters are estimated by using the Differential Evolution method [42] with the recommended parameter settings: crossover probability of 0.9, differential weight of 0.8, population size 50 and the rand/1/bin strategy. We set the number of evaluations of the objective function to 1000 times the number of constant model parameters. To assess the stability of the parameter estimator, we use 10 restarts of the Differential Evolution method. For simulating the reaction equations, ProBMoTs employs the Gillespie direct method [9] to obtain 20 realizations.

The parameter estimation method in ProBMoTs can use different objective functions for measuring the discrepancy between the realizations and the observed data. The first objective function we use in the experiments corresponds to a typical laboratory setting used in biology, where the measurements from multiple replicates of an experiment are averaged. Thus, the 20 realizations (*K* in the equation) are averaged just as the observed data:

(2) $$ \textit{RMSE}_{AR}(m) = \sum_{i} \frac{1}{\sqrt{N}} \lVert x_{i} - \widehat{x}_{i} \rVert, \quad \widehat{x}_{i} = \frac{1}{K} \sum_{k} \widehat{x}_{i}^{k},   $$

where *m* denotes the model, *i* iterates over the observed variables *x*_*i*_ and *k* iterates over the realizations, where $ \widehat {x}_{i}^{k} $ denotes the *k*-th realization of *x*_*i*_, and *N* is the number of observed time points.

Alternatively, in situations where the data are measured within a single experiment, we use the second objective function. Instead of averaging the realizations, we average the error of each realization, i.e.:

(3) $$ \textit{RMSE}_{SR}(m) = \frac{1}{K} \sum_{k} \sum_{i} \frac{1}{\sqrt{N}} \lVert x_{i} - \widehat{x}_{i}^{k} \rVert.   $$

Recall that the result of ProBMoTs is a list of models ranked with respect to their descending fit against the measured data, in our case, ascending model error. The trivial model selection strategy would be to select the model with the optimal value of the objective function. Note, however, that error-based estimates of model performance tend to overfit observations, a problem especially relevant in the context of noisy experimental data. To address the problem of overfitting, we use an alternative model selection approach that introduces a penalty for model complexity, measured as the number of reaction equations in the model. To additively combine the model complexity and the degree of fit into a single score, we normalize both to the [ 0,1] scale. The normalization is based on the minimal and maximal values of the degree of fit and complexity, respectively, over all the candidate model structures considered by ProBMoTs.

**Fig. 4:**
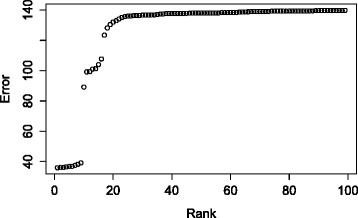
An example error profile of a ProBMoTs output that includes 100 models ranked according to increasing model error

We visualize the result of ProBMoTs (i.e., the ranked list of models) using an error profile, as depicted in Fig. 4. Each point of the error profile corresponds to a model induced by ProBMoTs and the y-axis of the profile corresponds to the respective value of the model selection criterion. In our experiments, we use the error profile to evaluate the ProBMoTs results in two ways. The first one selects the left-most model in the error profile, i.e., the model with the lowest model selection score, as the most appropriate model. We refer to this method as the singular method. This method is short-sighted since it only considers the best model. As an alternative to the singular method, we propose the inclusive method that considers models in the left-most plateau of the error profile. We employ a simple heuristic to identify plateaus: a relative change of error between two consecutive error-profile points that is above a threshold value of 0.1 indicates a plateau end. The first (leftmost) plateau of the error profile in Fig. 4 includes the cluster of ten points in the lower-left corner of the graph. Note that it includes ten top-ranked model structures that are indistinguishable in terms of the model error and therefore better represent the results of induction. The plateaus of the error profile lead to a partial ordering of the models.

Finally, to evaluate the results of induction, we compare the list of selected models to the list of plausible models *P* using a triple of metrics (recall, hit, plateau_size). The recall is the proportion of the plausible models in the first plateau of the error profile. The indicator hit tells us whether the first plateau contains the first model structure in *P*. The size of the plateau (plateau_size) indicates the discriminative power of the induction method: the smaller the plateau, the larger the discriminative power. The ideally performing induction method would lead to the triple (100 %, true, |*P*|).

## Results

In this section, we present the results of the evaluation of ProBMoTs on the four problems of inducing stochastic process-based models from knowledge and data. The first two are from the domain of gene regulatory networks, the other from the domain of epidemiology.

### Gene regulatory networks

We first address the task of modeling the simple gene regulatory network of the repressilator, introduced in the previous section. We select the model from Table 3 as a target model and set the list of plausible model structures *P* to contain a single structure that corresponds to the target model. We then perform two experiments. In the first, we assume that the kinetic rates in processes belonging to a single class of regulatory processes (degradation, translation and regulation) have the same values. To this end, we restructure the library of templates to introduce an global template entity that declares the global kinetic rates, which are then used by the process templates. In the second experiment, we perform induction without the assumption of global kinetic rates and therefore use the library of templates as presented in the previous section.

#### Global kinetic rates

The model of the repressilator considered here has been already addressed in other studies [6, 43]. Note, however, that both studies address only the task of parameter estimation from synthetic data assuming a single model structure. In our experiment, we also aim at identifying the structure of the model. We select the single model structure used in previous studies as our target and use the following values of the global kinetic rates: (*alpha*0,*alpha, beta, delta, n*)=(0.0,250.0,5.0,1.0,2.1). To obtain experimental data, we average 20 realizations of the target model in the time interval *t*∈[ 0,35]. Accordingly, we use the *RMSE*_*AR*_ objective function.

**Fig. 5:**
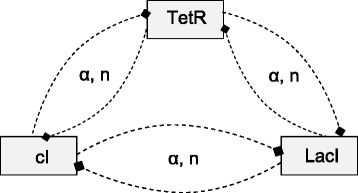
Graphical representation of the space of model structures considered during the induction of the repressilator model. Note that we do not assume fixed forms of the regulation interactions between the genes

In order to define a structure identification problem, we describe the space of possible model structures as represented in Fig. 5. Each rectangle represents a gene entity, while the dashed lines represent a regulation interaction between the entities. The interactions in the incomplete model are instantiated from the regulation process template from Table 1. This results in 3^6^=729 possible model structures, one of which is the target model structure of the repressilator.

**Fig. 6:**
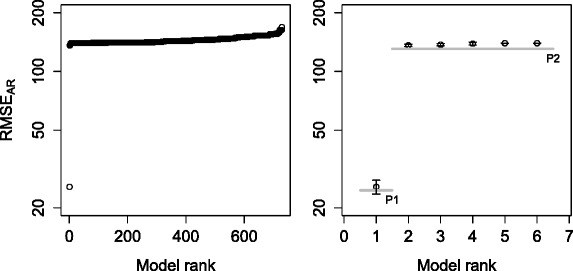
Error profile for the task of inducing the repressilator model with global kinetic rates. Complete error profile (*left*). The top six models in the first two plateaus with error bars showing the standard deviation across restarts. The gray horizontal lines depict plateaus (*right*)

Figure 6 depicts the error profile for the list of models obtained with ProBMoTs. First, note that the small standard deviations across the restarts of the parameter estimator show its stability. Furthermore, the first plateau of the error profile is easy to identify in the lower-left corner of the figure: it contains a single model. The structure of this model is a perfect match to the structure of the target model. Therefore, the recall is 100 %, the hit is true and the plateau size is 1, or in other words, the performance of ProBMoTs on this task is ideal. This result gives proof-of-principle evidence that confirms the ability of the developed process-based modeling method to induce both the structure and parameters of stochastic models from knowledge and data.

#### Local kinetic rates

To test the robustness of our method, we remove the assumption of global kinetic rates from the modeling scenario. Thus, we forget the changes we made to the library in the previous experiment and use the library as described in Table 1. Other than the different formalization of the domain knowledge, given the relaxed assumptions, the task remains the same: we use the same target model, the data set, the objective function (*RMSE*_*AR*_) and the list of plausible models as in the first experiment. The relaxed assumptions lead to an explosion in the parameter space, while the structure space remains the same. We want to test whether (and how) the relaxed modeling assumption will influence (deteriorate) the results of ProBMoTs.

**Fig. 7:**
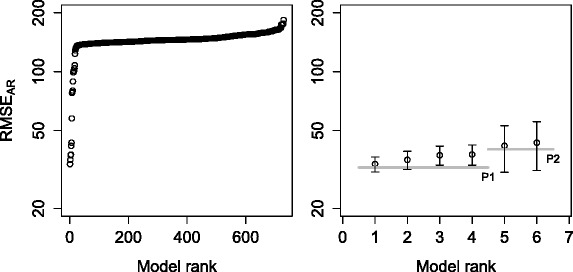
Error profile for the task of inducing the repressilator model with local kinetic rates. Complete error profile (*left*). The top six models in the first two plateaus with error bars showing the standard deviation across restarts. The gray horizontal lines depict plateaus (*right*)

**Fig. 8:**
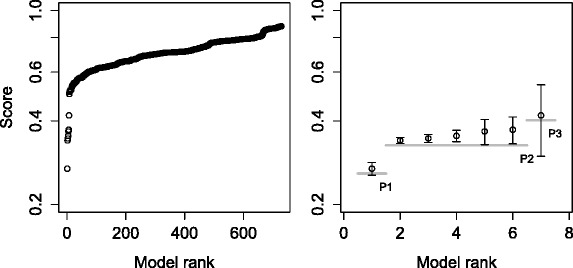
Error profile for the task of inducing the repressilator model with local kinetic rates based on a model selection score that takes into account model complexity penalization. Complete error profile (*left*). The top seven models in the first three plateaus with error bars showing the standard deviation across restarts. The gray horizontal lines depict plateaus (*right*)

The obtained error profile for the described task is shown in Fig. 7; note again the small standard deviation of the error over the parameter estimator restarts. The first plateau of the error profile includes four models. The second model has the structure that exactly matches the structure of the target model leading to the performance triple of (100 %, true, 4). The structures of the other three models in the plateau contain the repressilator motif and a number of additional gene regulation interactions, indicating an overfit of the experimental data. Indeed, Fig. 8 shows that if model complexity is taken into account when selecting models, the first plateau of the error profile includes only the target model, leading to the ideal performance triple of (100 %, true, 1).

### Compartmental epidemiological models

In the domain of epidemiology, we first formalize the knowledge to be used for establishing stochastic models, using the basic principles of compartmental modeling as presented by Brauer et al. [44]. There, the spread of disease is modeled by the flows of individuals between healthy and infected populations, referred to as compartments. Each flow is modeled using a reaction equation, where reactants and products correspond to compartments.

**Fig. 9:**
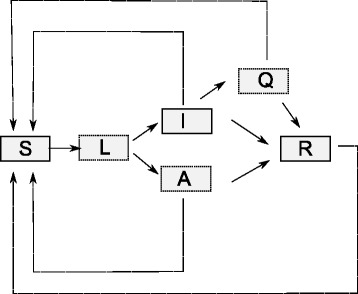
Graphical representation of a general compartmental model in epidemiology. The boxes correspond to compartments, i.e., subpopulations, and arrows denote the flows of individuals between compartments

Figure 9 graphically illustrates the general structure of epidemiological compartmental models. We distinguish between six compartments corresponding to six subpopulations of individuals that are susceptible (S) to the observed disease, latently infected (L), infected with (I) and without symptoms (A, i.e., asymptomatic), quarantined (Q) and recovered (or removed, in case of fatal diseases, R). In the library of modeling knowledge, all these compartments are represented with a single entity template compartment which has the variable property of noi, representing the number of individuals in the compartment at a given time point.

At the point of introduction of a pathogen in the population, the entire population can be considered to consist of susceptible individuals (in the compartment S), except for the individuals by whom the pathogen is introduced. From this point on, we can observe different processes of flow between compartments. One way to model the infection of individuals is to assume that all infected individuals manifest the disease symptoms. In this case, the A compartment is not populated. An alternative, more complex, model assumes that we can also have infected individuals that do not manifest the symptoms. In both cases, the infection might cause a direct flow from S to I (and/or A) or indirect flow through the L compartment of latently infected individuals.

The recovery of individuals from a disease can either cause flows from the A and I compartments to the population of recovered (or removed) individuals R or cause flows from the A and I to the population of susceptible individuals S. In any case, the recovery of the individuals from I can be controlled by moving the infected individuals to the quarantine compartment Q. Finally, the general model involves a flow of individuals from the recovery compartment to the population of susceptible individuals.

The general model can be instantiated to a number of variants, ranging from the simple SIR model that assumes only three compartments of susceptible, infected and recovered individuals, through the SLIR model that introduces the population of latently infected individuals, to the most complex SLIAQRS model that comprises all the compartments depicted in Fig. 9. For example, the SIR model includes two processes. The first instantiates the template process of infection_symptomatic that includes a single reaction equation: S.noi + I.noi → I.noi + I.noi [i], where *i* represents the rate of infection. The other process represents the template of recovery_symptomatic that includes the reaction equation I.noi → R.noi [r], where *r* denotes the recovery rate.

In contrast to the previous synthetic tasks, here we use two data sets of real measurements for induction. These come from two epidemic outbreaks, the outbreak of the Great Plague in Eyam in 1666 [45] and the outbreak of influenza type A subtype H3N2 in Tristan da Cunha in 1967 [46, 47]. The measurements for the case of the outbreak in Eyam are taken bimonthly at seven time points in the period from 3rd of July to 20th of October 1666. They include two variables: number of healthy individuals and the number of individuals that have complained of symptoms. The measurements from Tristan da Cunha are taken daily at 21 time points in October 1967. They also include two variables: number of individuals showing symptoms of infection and the number of recovered individuals.

To match the compartment variables to the variables in the data sets, we calculate the number of healthy (individuals not showing any symptoms of infection) as the sum of the number of individuals in the S, L and A compartments, the number of infected as the sum of the number of individuals in the I and Q compartments and the number of recovered as the number of individuals in the R compartment.

In accordance with the experimental setting for obtaining the measurements, we use the second objective function *RMSE*_*SR*_. Since the experimental data comes from real and therefore noisy measurements, we take into account model complexity to obtain the model selection score.

#### Eyam plague outbreak

For this task, we consider all possible instances of the general model as previously described, by introducing a small set of constraints of mutual exclusivity of symptomatic and asymptomatic infection, thus instantiating only the corresponding recovery for each type of infection. The total number of model structures under these constraints is 24. The initial conditions at the first time point were set to 254 individuals in the S, 7 individuals in the I and 0 in the other compartments, which exactly matches the initial conditions from the original study by Ragget [45]. The same paper proposes two plausible model structures: SIR, the structure that has been analyzed in the paper, and SLIR, suggested as the most promising one for further study. Thus, our list of plausible models structures *P* is (SIR, SLIR).

**Fig. 10:**
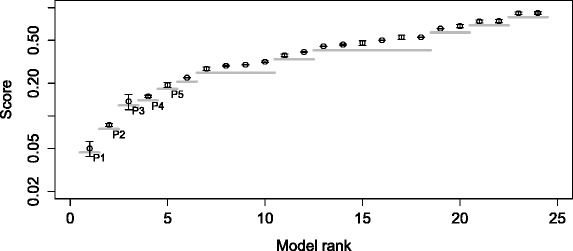
Error profile for the Eyam plague modeling task. The error bars show the standard deviation of the model selection score across the runs. The gray horizontal lines depict plateaus

The first plateau of the error profile, depicted in Fig. 10, contains a single model that has the SIR structure. Therefore the recall is 50 %, the hit is true and the plateau size is 1. The model with the SLIR structure is ranked as second and comprises the second error-profile plateau. Thus, when considering the two models in the two left-most plateaus, ProBMoTs successfully reconstructs the two plausible model structures suggested before [45]. Note that the complexity-based model selection score bears high discriminative power, since each model forms its own plateau. The next four plateaus of the error profile include the SIRS, SLIRS, SIQR and SLIQR models, which render model structures that extend the basic SIR and SLIR with the assumptions of survivors (return to the susceptible compartment) or a quarantine compartment to provide plausible explanations of the observed data.

#### Tristan da Cunha influenza outbreak

For this task, we consider the same set of 24 model structures that instantiate the general model from Fig. 9. Based on the data available, we set the initial number of infected individuals to 1, other initial values to 0, except for the initial number of susceptible individuals that was fitted as a model parameter. We selected the two best performing model structures from Toni et al. [6] as plausible and set *P* to (SLIR, SIR). The other two model structures considered in the study are a modified SLIR structure, that includes time-delayed flow models, and a SIRS structure.

**Fig. 11:**
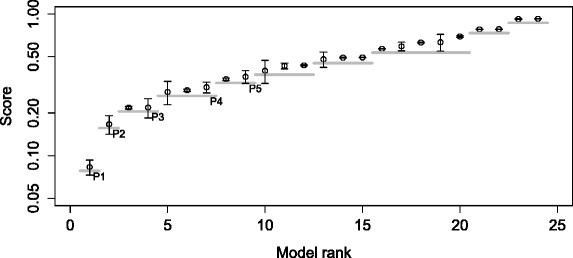
Error profile for the Tristan da Cunha influenza outbreak modeling task. The error bars show the standard deviation of the model selection score across the runs. The gray horizontal lines depict plateaus

The first plateau of the error profile, depicted in Fig. 11, contains the SLIR model that is the first model in *P*, leading to a recall of 50 %, the hit indicator is true and the plateau size is 1. The second ranked model in the second plateau has the SIR structure of the second model in *P*. As in the case of the Eyam plague experiments, ProBMoTs perfectly reconstructed the results of the previous modeling experiments reported by Toni et al. [6].

## Discussion

The formalism for stochastic process-based modeling, that we introduce in this work, retains the modular and straight-forward specification of entire classes of model structures from its deterministic counterpart. In contrast to the formalisms commonly used in systems biology that employ different levels of abstraction but focus primarily on the efficient description of a single model structure [10–13], the process-based formalism allows for describing uncertainty in both the structure and parameter values of a model by representing classes of model structures. The introduction of reaction-equation based description of processes improves the understandability of process-based models and allows for their stochastic interpretation, improving the generality and utility of the process-based modeling approach and bringing it closer to the domain of biology.

The experimental evaluation shows that our approach can be successfully applied to a range of problems of learning stochastic models. These can come from different biological domains and represent phenomena at different scales. Our approach exhibits excellent performance on the considered tasks, producing accurate and understandable models and successfully reconstructing the results of previous modeling efforts. The proposed approach can be applied to an arbitrary domain of interest by encoding an appropriate library of template entities and processes encountered in the particular domain.

However, several limiting issues may arise during the application of the proposed approach.

First, solving the parameter estimation task for each model structure can lead to both identifiability and distinguishability problems. The identifiability of the model parameters is a problem often encountered when modeling biological systems [48]. Performing identifiability analysis for each candidate structure is in principle possible. However, this can be challenging in terms of computational complexity when considering a large number of candidate model structures. A more tractable problem is the one of distinguishability of the candidate model structures in terms of the applied model selection criteria. Within process-based modeling, this problem presents itself in the form of long plateaus in the error profile. This problem has been studied for the task of learning deterministic models of dynamical systems from data and domain knowledge [32]. The study shows that the problem of distinguishability can be successfully addressed by the introduction of problem specific, domain dependent criteria for parameter optimization and model selection. Although this study is limited to the case of deterministic models, further work can extend its scope to stochastic modeling.

Second, the encoding of very large and complex systems in the proposed formalisms may be cumbersome. The use of reaction equations to encode a system with many entities, that is comprised of a large number of simple and repeating interactions might lead to excessively lengthy descriptions. Following concepts from related work, the issue of combinatorial complexity of composing models from elementary interactions may be solved by rule- or agent-based formalisms by introducing further abstraction.

Finally, the third limitation of our approach is related to computational cost. The simulation of a candidate model is the computationally most expensive step of the process of model induction. Therefore, the computational cost is proportional to the number of evaluations (and the number of simulations per evaluation) needed for each model during the parameter estimation task. Our method requires exhaustive enumeration and optimization of a number of candidate model structures defined by entity/process templates organized in multiple-level hierarchies of alternatives within a library of domain knowledge. Subsequently, a combinatorial explosion is possible if the problem is not well constrained. This is exactly why the process-based modeling approach includes the facility for imposing constraints on the space of possible model structures by allowing for the definition of an incomplete model.

## Conclusion

The area of computational biology lacks a unified methodology for modeling dynamical systems that would include a formalism for representing complex dynamics in a manner easily understandable to biologists and modeling experts. In this paper, we advocate the use of process-based modeling for this purpose. It allows for understandable description of a space of candidate model structures for a given modeling task. It allows for both deterministic and stochastic interpretation of process-based models. Also, it allows for automated induction of models from data and knowledge.

In order to bridge the gap between the existing and commonly used tools for modeling the dynamics of biological systems and the machine learning approaches to computational scientific discovery, we have extended the scope of process-based modeling approaches, specifically ProBMoT, to include stochastic models. As an intermediate representation, our ProBMoTs formalism includes the finer, more intuitive and easier to comprehend representation of reaction equations, which should increase the ease of use of process-based modeling in biology. This finer-grained representation of processes is a feature that broadens the possibilities of interpretation, mainly in the direction of capturing the inherent stochasticity of dynamical systems in biology.

Through the tasks considered in this work, we have shown that our approach can deal with complex parameter and structure search spaces, in lightly constrained settings, with synthetically generated tasks and in less constrained real world problems. We have thus demonstrated the potential of our approach for automated discovery of novel scientific knowledge in domains that require stochastic modeling of dynamical systems. Our results also point at an array of possibilities for further evaluation and improvement.

The presented extension of the process-based formalism integrates reaction equations as a proxy that allows for multiple interpretations of the process-based models. However, we can continue this initial step by integrating other higher-level formalisms. Combining rule-based modeling languages with the process templates from the process-based modeling formalism can be considered as a first direction for further work. The introduction of rule-based constraints would allow for automated modeling of more complex systems.

Another direction for further work stems naturally from the formulation of the modeling task as a combinatorial search problem. It concerns the implementation of incomplete, heuristics-based search strategies over the space of candidate models. Although a comparative evaluation with the method using exhaustive search is needed to establish its utility, this extension will scale-up our approach towards applications to large-scale modeling problems.

Other factors might also contribute to the overall success of our approach, e.g., the choice of a parameter estimation method and a method for simulation. Existing literature offers comparisons of the performance of different parameter estimation methods on single model structures for modeling tasks from the domain of systems biology (both deterministic and stochastic) [49, 50]. A comparison of the performance of different parameter estimation methods has also been performed in the context of deterministic process-based modeling of aquatic ecosystems [40]. The conclusions from these studies are a good starting point to investigate their performance in the context of stochastic process-based modeling tasks.

## Ethics approval

No aspect of this study required ethics approval.

## Availability of data and materials

The libraries of domain knowledge, incomplete models and data supporting the conclusions of this article are available in the Zenodo repository https://zenodo.org/record/45503 (doi:n).

## Endnotes

^1^ The notions of entities and processes are ontologically well-grounded and are present (as continuants and occurrents) in the Basic Formal Ontology.

^2^ ProBMoT and ProBMoTs are released under the terms of the BSD license http://probmot.ijs.si/licence.html.
